# Supplementary material for: Novel neuroanatomical integration and scaling define avian brain shape evolution and development
Source: eLife. 2021 Jul 6;10:e68809. doi: 10.7554/eLife.68809 (PMC8260227; doi:10.7554/eLife.68809)
Supplement: Supplementary file 1. — (a) List of taxa sampled for this study, with the exclusion of Alligator and Gallus. Institutional abbreviations: AMNH, American Museum of Natural History, New York, NY, USA; BMNH, British Museum of Natural History, London, UK; FMNH, Field Museum of Natural History, Chicago, IL, USA; KU, University of Kansas, Lawrence, KS, USA; NMNH, National Museum of Natural History, Washington DC, USA; TCWC, Texas Cooperative Wildlife Collection, College Station, TX, USA; TMM, Texas Memorial Museum, Austin, TX, USA; WDC, Wyoming Dinosaur Center, Thermopolis, WY, USA. (b) List of discrete landmarks and density of semi-landmarks for each neuroanatomical region. (c) Phylogenetic signal (Blomberg’s K), allometry, and evolutionary allometry in endocranial shape. Results generated using physignal, procD.lm, procD.pgls functions in geomorph R package v3.2.1 (Adams and Otárola-Castillo, 2013). Results from analysis on globally and locally aligned regions are presented as first and second values within a cell, respectively. Allometry evaluated with log-transformed centroid size of the entire endocast and local region, respectively. *, **, and *** indicate p<0.05, <0.01, and <0.001, respectively, based on 1000 pseudo-replications. (d) Integration within and between locally aligned neuroanatomical regions. Degree of integration is measured by correlation coefficient from two-block partial least-squares analysis (RPLS; upper off-diagonal) and correlation coefficient (ρ; diagonal, lower off-diagonal) using the R packages geomorph v3.2.1 (Adams and Otárola-Castillo, 2013) and EMMLiv2 v0.0.3 (Goswami and Finarelli, 2016), respectively. Interspecific analyses are phylogenetically corrected using phylogenetic generalized least-squares method. (e) Integration within and among globally aligned neuroanatomical regions. The degree of integration is measured by correlation coefficient from two-block partial least-squares analysis (RPLS; upper off-diagonal) and correlation coefficient (ρ; diagonal, low [file elife-68809-supp1.docx]

**Novel neuroanatomical integration and scaling define avian brain shape evolution and development.**

Akinobu Watanabe, Amy M. Balanoff, Paul M. Gignac, M. Eugenia L. Gold, Mark A. Norell

**Supplementary File 1**

**1a.** **List of taxa sampled for this study, with the exclusion of *Alligator* and *Gallus*.** Institutional abbreviations: **AMNH,** American Museum of Natural History, New York, NY, USA; **BMNH,** British Museum of Natural History, London, UK; **FMNH,** Field Museum of Natural History, Chicago, IL, USA; **KU,** University of Kansas, Lawrence, KS, USA; **NMNH,** National Museum of Natural History, Washington DC, USA; **TCWC,** Texas Cooperative Wildlife Collection, College Station, TX, USA; **TMM,** Texas Memorial Museum, Austin, TX, USA; **WDC,** Wyoming Dinosaur Center, Thermopolis, WY, USA.

| Taxonomic name | Specimen no. |  |
| --- | --- | --- |
| *Alioramus altai* | IGM 100/1844 |  |
| *Citipati osmolskae* | IGM 100/973 |  |
| *Incisivosaurus gauthieri* | IVPP V 13326 |  |
| Unnamed troodontid | IGM 100/1126 |  |
| *Khaan mckennai* | IGM 100/973 |  |
| *Zanabazar junior* | IGM 100/1 |  |
| *Alca torda* | AMNH 17532 |  |
| *Anas platyrhynchos* | TMM M-uncat. |  |
| *Apteryx* sp. | TMM M-uncat. |  |
| *Archaeopteryx lithographica* | WDC CSG 100 |  |
| *Brotogeris chrysopteris* | FMNH 330249 |  |
| *Bucorvus abyssinicus* | TMM M-5946 |  |
| *Caloenas nicobarica* | NMNH 19715 |  |
| *Casuarius casuarius* | AMNH 3200 |  |
| *Casuarius unappendiculatus* | AMNH 2729 |  |
| *Chauna chavaria* | KU 81969 |  |
| *Chordeiles minor* | TMM M-uncat. |  |
| *Coragyps atratus* | TMM M-uncat. |  |
| *Crypturellus tataupa* | AMNH 8560 |  |
| *Diomedea* sp. | TMM M-uncat. |  |
| *Dromaius novaehollandiae* | AMNH 11709 |  |
| *Eudyptes* sp. | TMM M-uncat. |  |
| *Fregata magnificens* | FMNH 37858 |  |
| *Gallirallus australis* | NMNH 19021 |  |
| *Gallirallus rovianae* | AMNH 30329 |  |
| *Gavia immer* | TCWC 13.300 |  |
| *Grus canadensis* | TMM M-uncat. |  |
| *Haliaeetus leucocephalus* | TMM M-7260 |  |
| *Melanerpes aurifrons* | FMNH 108742 |  |
| *Nestor meridionalis* | AMNH 27323 |  |
| *Phaethon rubricauda* | FMNH 346039 |  |
| *Phalacrocorax harrisi* | AMNH 2312 |  |
| *Phalacrocorax penicillatus* | TMM M-1180 |  |
| *Pinguinus impennis* | AMNH 261 |  |
| *Podilymbus podiceps* | TMM M-7139 |  |
| *Ptilinopus melanospila* | TMM M-uncat. |  |
| *Gallirallus philippensis* | AMNH 3442 |  |
| *Raphus cucullatus* | NHMUK A9040 |  |
| *Rhea americana* | AMNH 6470 |  |
| *Cariama cristata* | AMNH 8604 |  |
| *Struthio camelus* | AMNH 3199 |  |
| *Tachyeres brachypterus* | NMNH 555468 |  |
| *Tachyeres leucocephalus* | AMNH 8513 |  |
| *Tachyeres pteneres* | NMNH 490937 |  |

**1b. List of discrete landmarks and density of semi-landmarks for each neuroanatomical region.**

| Region | Landmark density | Discrete landmarks |
| --- | --- | --- |
| Left/right cerebrum | 54 | Anterior tip of the cerebrum on dorsal side. |
|  |  | Posteromedial point of the left/right cerebrum on dorsal side. |
|  |  | Dorsal-most junction point of cerebrum and optic lobe. |
|  |  | Ventral-most junction point of cerebrum and optic lobe. |
| Left/right optic lobe | 54 | Dorsal-most junction point of cerebrum and optic lobe. |
|  |  | Ventral-most junction point of cerebrum and optic lobe. |
|  |  | Junction point of optic lobe, midbrain, and medulla. |
|  |  | Junction of optic lobe, cerebellum, and medulla. |
| Cerebellum | 30 | Anterior-most median point of cerebellum on dorsal side. |
|  |  | Left and right anteroventral points of the cerebellum. |
|  |  | Left and right dorsal points at the base of flocculus. |
|  |  | Left and right posterolateral points of the cerebellum. |
|  |  | Posterior-most median point of the cerebellum on dorsal side. |
| Medulla | 29 | Anterior-most median point adjacent to midbrain on ventral side. |
|  |  | Left and right junctions of optic lobe and medulla. |
|  |  | Left and right posterolateral points of medulla. |
|  |  | Posterior-most median point of medulla. |

**1c. Phylogenetic signal (Blomberg’s *K*), allometry, and evolutionary allometry in endocranial shape.** Results generated using physignal, procD.lm, procD.pgls functions in geomorph R package v3.2.1 (Adams and Otárola-Castillo, 2013). Results from analysis on globally and locally aligned regions are presented as first and second values within a cell respectively. Allometry evaluated with log-transformed centroid size of the entire endocast and local region respectively. ^*^, ^**^, and ^***^ indicate *P* < 0.05, < 0.01, and <0.001 based on 1,000 pseudo-replications.

| Dataset | Region | Phylogenetic signal (*K*) | Allometry (R^2^) | Evolutionary allometry (R^2^) |
| --- | --- | --- | --- | --- |
| Archosauria^1^ | overall | 0.074^***^ | 0.122^***^ | 0.039 |
|  | cerebrum | 0.084^***^ / 0.044^**^ | 0.100^*^ / 0.034 | 0.034 / 0.371^**^ |
|  | optic lobe | 0.082^***^ / 0.027^*^ | 0.135^**^ / 0.054^*^ | 0.049 / 0.025 |
|  | cerebellum | 0.057^**^ / 0.025 | 0.087^*^ / 0.161^**^ | 0.039 / 0.011 |
|  | medulla | 0.070^**^ / 0.018 | 0.192^***^ / 0.242^***^ | 0.038 / 0.656^***^ |
| non-avialan | overall | 0.814 | 0.242 | 0.242 |
| Coelurosauria | cerebrum | 0.761 / 0.680 | 0.140 / 0.111 | 0.105 / 0.094 |
|  | optic lobe | 0.751 / 0.753 | 0.234 / 0.234 | 0.224 / 0.195 |
|  | cerebellum | 0.975^*^ / 0.972^*^ | 0.239 / 0.230 | 0.205 / 0.214 |
|  | medulla | 0.754 / 0.673 | 0.375 / 0.680^**^ | 0.437^*^ / 0.724^**^ |
| Neornithes | overall | 0.035^*^ | 0.093^**^ | 0.048 |
|  | cerebrum | 0.041^*^ / 0.027 | 0.081^*^ / 0.065^*^ | 0.043 / 0.427^**^ |
|  | optic lobe | 0.041^*^ / 0.019 | 0.131^***^ / 0.034 | 0.057 / 0.033 |
|  | cerebellum | 0.033 / 0.021 | 0.029 / 0.076^*^ | 0.046 / 0.016 |
|  | medulla | 0.023 / 0.015 | 0.171^**^ / 0.188^**^ | 0.051 / 0.720^**^ |
| *Alligator* development | overall | — | 0.414^***^ | — |
|  | cerebrum | — | 0.384^***^ / 0.356^**^ | — |
|  | optic lobe | — | 0.391^***^ / 0.403^***^ | — |
|  | cerebellum | — | 0.375^***^ / 0.265^**^ | — |
|  | medulla | — | 0.523^***^ / 0.499^***^ | — |
| *Gallus* development | overall | — | 0.446^***^ | — |
|  | cerebrum | — | 0.457^***^ / 0.399^***^ | — |
|  | optic lobe | — | 0.497^***^ / 0.250^***^ | — |
|  | cerebellum | — | 0.329^***^ / 0.335^***^ | — |
|  | medulla | — | 0.472^***^ / 0.316^**^ | — |

**^1^** excludes developmental series of *Alligator* and *Gallus* but includes their largest specimens.

**1d. Integration within and between locally aligned neuroanatomical regions.** Degree of integration is measured by correlation coefficient from two-block partial least squares analysis (R_PLS_; upper off-diagonal) and correlation coefficient (ρ; diagonal, lower off-diagonal) using the R packages geomorph v3.2.1 (Adams and Otárola-Castillo, 2013) and EMMLiv2 v0.0.3 (Goswami and Finarelli, 2016) respectively. Interspecific analyses are phylogenetically corrected using phylogenetic generalized least-squares method.

Archosauria

|  | Cerebrum | Optic Lobe | Cerebellum | Medulla |
| --- | --- | --- | --- | --- |
| Cerebrum | 0.36 | 0.991 | 0.989 | 0.993 |
| Optic Lobe | 0.15 | 0.31 | 0.993 | 0.997 |
| Cerebellum | 0.18 | 0.15 | 0.36 | 0.994 |
| Medulla | 0.10 | 0.10 | 0.11 | 0.38 |

non-avialan Coelurosauria

|  | Cerebrum | Optic Lobe | Cerebellum | Medulla |
| --- | --- | --- | --- | --- |
| Cerebrum | 0.41 | 0.989 | 0.654 | 0.916 |
| Optic Lobe | 0.25 | 0.33 | 0.773 | 0.888 |
| Cerebellum | 0.19 | 0.19 | 0.37 | 0.936 |
| Medulla | 0.17 | 0.20 | 0.29 | 0.50 |

Neornithes

|  | Cerebrum | Optic Lobe | Cerebellum | Medulla |
| --- | --- | --- | --- | --- |
| Cerebrum | 0.35 | 0.992 | 0.990 | 0.994 |
| Optic Lobe | 0.11 | 0.28 | 0.993 | 0.997 |
| Cerebellum | 0.15 | 0.11 | 0.36 | 0.994 |
| Medulla | 0.10 | 0.11 | 0.11 | 0.37 |

*Alligator* development

|  | Cerebrum | Optic Lobe | Cerebellum | Medulla |
| --- | --- | --- | --- | --- |
| Cerebrum | 0.41 | 0.808 | 0.625 | 0.688 |
| Optic Lobe | 0.20 | 0.32 | 0.728 | 0.913 |
| Cerebellum | 0.16 | 0.16 | 0.35 | 0.652 |
| Medulla | 0.17 | 0.27 | 0.20 | 0.52 |

*Gallus* development

|  | Cerebrum | Optic Lobe | Cerebellum | Medulla |
| --- | --- | --- | --- | --- |
| Cerebrum | 0.38 | 0.966 | 0.910 | 0.823 |
| Optic Lobe | 0.18 | 0.31 | 0.943 | 0.840 |
| Cerebellum | 0.20 | 0.17 | 0.33 | 0.942 |
| Medulla | 0.17 | 0.16 | 0.21 | 0.37 |

**1e.**  **Integration within and among globally aligned neuroanatomical regions.** The degree of integration is measured by correlation coefficient from two-block partial least squares analysis (R_PLS_; upper off-diagonal) and correlation coefficient (ρ; diagonal, lower off-diagonal) using the R packages geomorph v3.2.1 (Adams and Otárola-Castillo, 2013) and EMMLiv2 v0.0.3 (Goswami and Finarelli, 2016) respectively. Interspecific analyses are phylogenetically corrected using phylogenetic generalized least-squares method.

Archosauria

|  | Cerebrum | Optic Lobe | Cerebellum | Medulla |
| --- | --- | --- | --- | --- |
| Cerebrum | 0.43 | 0.990 | 0.987 | 0.991 |
| Optic Lobe | 0.28 | 0.59 | 0.995 | 0.995 |
| Cerebellum | 0.27 | 0.35 | 0.60 | 0.994 |
| Medulla | 0.20 | 0.29 | 0.24 | 0.67 |

non-avialan Coelurosauria

|  | Cerebrum | Optic Lobe | Cerebellum | Medulla |
| --- | --- | --- | --- | --- |
| Cerebrum | 0.46 | 0.972 | 0.877 | 0.867 |
| Optic Lobe | 0.36 | 0.47 | 0.833 | 0.808 |
| Cerebellum | 0.26 | 0.30 | 0.65 | 0.913 |
| Medulla | 0.23 | 0.35 | 0.43 | 0.55 |

Neornithes

|  | Cerebrum | Optic Lobe | Cerebellum | Medulla |
| --- | --- | --- | --- | --- |
| Cerebrum | 0.39 | 0.991 | 0.989 | 0.992 |
| Optic Lobe | 0.25 | 0.47 | 0.995 | 0.997 |
| Cerebellum | 0.20 | 0.21 | 0.49 | 0.996 |
| Medulla | 0.17 | 0.26 | 0.22 | 0.64 |

*Alligator* development

|  | Cerebrum | Optic Lobe | Cerebellum | Medulla |
| --- | --- | --- | --- | --- |
| Cerebrum | 0.50 | 0.917 | 0.927 | 0.873 |
| Optic Lobe | 0.27 | 0.46 | 0.911 | 0.929 |
| Cerebellum | 0.29 | 0.26 | 0.45 | 0.866 |
| Medulla | 0.15 | 0.31 | 0.24 | 0.51 |

*Gallus* development

|  | Cerebrum | Optic Lobe | Cerebellum | Medulla |
| --- | --- | --- | --- | --- |
| Cerebrum | 0.43 | 0.967 | 0.973 | 0.824 |
| Optic Lobe | 0.31 | 0.45 | 0.965 | 0.893 |
| Cerebellum | 0.26 | 0.28 | 0.46 | 0.929 |
| Medulla | 0.30 | 0.38 | 0.23 | 0.57 |

**1f. Integration between neuroanatomical regions using covariation ratios (CR)** (Adams, 2016)**.** Degree of integration between globally aligned regional shapes are listed in the upper off-diagonal elements and that of locally aligned regional shapes in the lower off-diagonal elements. Interspecific analyses are phylogenetically corrected using phylogenetic generalized least-squares method.

Archosauria

|  | Cerebrum | Optic Lobe | Cerebellum | Medulla |
| --- | --- | --- | --- | --- |
| Cerebrum |  | 0.861 | 0.709 | 0.944 |
| Optic Lobe | 0.899 |  | 0.866 | 0.921 |
| Cerebellum | 0.694 | 0.733 |  | 0.758 |
| Medulla | 0.883 | 0.959 | 0.796 |  |

non-avialan Coelurosauria

|  | Cerebrum | Optic Lobe | Cerebellum | Medulla |
| --- | --- | --- | --- | --- |
| Cerebrum |  | 1.014 | 0.907 | 0.823 |
| Optic Lobe | 0.991 |  | 0.905 | 0.825 |
| Cerebellum | 0.633 | 0.774 |  | 0.949 |
| Medulla | 0.739 | 0.858 | 1.006 |  |

Neornithes

|  | Cerebrum | Optic Lobe | Cerebellum | Medulla |
| --- | --- | --- | --- | --- |
| Cerebrum |  | 0.877 | 0.816 | 0.889 |
| Optic Lobe | 0.912 |  | 0.951 | 0.981 |
| Cerebellum | 0.797 | 0.891 |  | 0.944 |
| Medulla | 0.925 | 0.998 | 0.925 |  |

*Alligator* development

|  | Cerebrum | Optic Lobe | Cerebellum | Medulla |
| --- | --- | --- | --- | --- |
| Cerebrum |  | 0.861 | 0.877 | 0.781 |
| Optic Lobe | 0.791 |  | 0.884 | 0.920 |
| Cerebellum | 0.538 | 0.696 |  | 0.833 |
| Medulla | 0.625 | 0.902 | 0.581 |  |

*Gallus* development

|  | Cerebrum | Optic Lobe | Cerebellum | Medulla |
| --- | --- | --- | --- | --- |
| Cerebrum |  | 0.973 | 0.916 | 0.792 |
| Optic Lobe | 0.908 |  | 0.910 | 0.890 |
| Cerebellum | 0.900 | 0.859 |  | 0.865 |
| Medulla | 0.781 | 0.746 | 0.971 |  |

**1g.** Comparison of integration among neuroanatomical regions using the compare.pls function in the geomorph R package (Adams and Otárola-Castillo, 2013; Adams and Collyer, 2016). “+” and “­­–” denote greater and lesser integration in Neornithes and *Gallus* compared to non-avialan coelurosaurs and *Alligator*, respectively. Integration among species calculated upon phylogenetic correction.  ^*^, ^**^, and ^***^ indicate *P* < 0.05, < 0.01, and <0.001 (one-tailed) based on 1,000 pseudo-replications. Number preceding and following “/” indicate results based on globally and locally aligned data, respectively.

Neornithes relative to Non-Avialan Coelurosauria (overall: +^***^ / +^***^)

|  | Cerebrum | Optic Lobe | Cerebellum | Medulla |
| --- | --- | --- | --- | --- |
| Cerebrum |  |  |  |  |
| Optic Lobe | +^***^ / +^***^ |  |  |  |
| Cerebellum | +^***^ / +^***^ | +^***^ / +^***^ |  |  |
| Medulla | +^***^ / +^***^ | +^***^ / +^***^ | +^***^ / +^***^ |  |

*Gallus* relative to *Alligator* (overall: + / +)

|  | Cerebrum | Optic Lobe | Cerebellum | Medulla |
| --- | --- | --- | --- | --- |
| Cerebrum |  |  |  |  |
| Optic Lobe | + / + |  |  |  |
| Cerebellum | + / +^*^ | + / + |  |  |
| Medulla | ^­^– / + | – / – | + / +^*^ |  |
